# Hybrid EEG-fNIRS BCI Fusion Using Multi-Resolution Singular Value Decomposition (MSVD)

**DOI:** 10.3389/fnhum.2020.599802

**Published:** 2020-12-08

**Authors:** Muhammad Umer Khan, Mustafa A. H. Hasan

**Affiliations:** Department of Mechatronics Engineering, Atilim University, Ankara, Turkey

**Keywords:** hybrid BCI, fNIRS, EEG, multi-resolution singular value decomposition, multi-modal fusion, channel selection, classification

## Abstract

Brain-computer interface (BCI) multi-modal fusion has the potential to generate multiple commands in a highly reliable manner by alleviating the drawbacks associated with single modality. In the present work, a hybrid EEG-fNIRS BCI system—achieved through a fusion of concurrently recorded electroencephalography (EEG) and functional near-infrared spectroscopy (fNIRS) signals—is used to overcome the limitations of uni-modality and to achieve higher tasks classification. Although the hybrid approach enhances the performance of the system, the improvements are still modest due to the lack of availability of computational approaches to fuse the two modalities. To overcome this, a novel approach is proposed using Multi-resolution singular value decomposition (MSVD) to achieve system- and feature-based fusion. The two approaches based up different features set are compared using the KNN and Tree classifiers. The results obtained through multiple datasets show that the proposed approach can effectively fuse both modalities with improvement in the classification accuracy.

## 1. Introduction

The brain-computer interface (BCI) provides an interlink between the brain and external devices (Vidal, 1973; Wolpaw et al., 2002). The information received from the brain in the form of physiological/magnetic/metabolic signals is decoded and interpreted to determine the user intentions, and is later utilized for various purposes, such as rehabilitation (Do et al., 2013; Khan R. A. et al., 2018); control of robots (Doud et al., 2011; Bozinovski, 2016; Khan A. H. et al., 2018; Rosca et al., 2018; Duan et al., 2019) and of prosthetics (Buch et al., 2018; Yanagisawa et al., 2019); and neurogaming (Paszkiel, 2016, 2020; Vasiljevic and de Miranda, 2020). Among the existing non-invasive acquisition methods, arguably EEG (Wolpaw et al., 2002; Pfurtscheller et al., 2006; Choi, 2013; Abiri et al., 2019) and fNIRS (Ferrari et al., 1985; Delpy et al., 1988; Coyle et al., 2004, 2007; Fazli et al., 2012; Naseer and Keum-Shik, 2015; Yin et al., 2015) are considered the most explored. EEG is the physiological method, with low spatial and high temporal resolution, that measures the brain activity in the form of electrical impulses (volts) using the electrodes placed at specific positions on the scalp. On the other hand, fNIRS, based upon metabolic signals, measures the level of oxygenation and de-oxygenation in the blood with high spatial and low temporal resolution. Due to low temporal resolution, fNIRS may require several seconds to monitor the blood levels (Khan and Hong, 2017). The time involved in monitoring causes a delay in generating execution commands. For the fNIRS, this duration is almost 9 times that of EEG (Khan and Hong, 2017). Additionally, in comparison to EEG, fNIRS is considered more robust against electromyogram artifacts and electrical noises (Blankertz et al., 2010; Ahn and Jun, 2015, 2017). The limitations of both modalities led to a multi-modal system, known as the hybrid EEG-fNIRS BCI, that has the ability to overcome the drawbacks of uni-modal systems and to improve the performance.

The hybrid EEG-fNIRS BCI has attracted the attention of many researchers due to its mobility, cost-effectiveness, and enhanced information content (compared to the uni-modal). Since the EEG obtains information from the physiological signals, and fNIRS uses metabolic signals to detect the hemodynamic, there is no significant interference between the two modalities. This further helps to obtain an enhanced BCI performance. The first notable study that concurrently recorded EEG-fNIRS data to perform motor imagery tasks was done by Fazli et al. (2012). The authors reported an improvement in the classification accuracy by 5% on average when compared to the single modality. After the promising results obtained by Fazli et al. (2012), more researchers tried to utilize the hybrid BCI, either to increase the classification accuracy and/or to generate more control commands (Khan et al., 2014; Koo et al., 2015; Aghajani et al., 2017; Ge et al., 2017; Shin et al., 2018a). The most explored areas where the hybrid BCI is utilized include mental stress (Al-Shargie et al., 2016; Aghajani et al., 2017) and gait rehabilitation (Berger et al., 2019), among many others (Putze et al., 2014; Zama et al., 2019). Though the hybrid EEG-fNIRS BCI has been able to upheld its supremacy against single modality both in terms of accuracy and stability, there are still some challenges related to the integration of both modalities.

Data-fusion in multi-modality is a challenging problem since the brain imaging data is different in nature, thus making the analysis more difficult. Most of the previous studies focused on feature-based fusion through concatenating EEG and fNIRS features (Putze et al., 2014; Hong et al., 2018; Shin et al., 2018a), and by providing them the support of other power tools. Joint independent component analysis (jICA), which was previously used for integrating EEG and fMRI (Calhoun and Adali, 2008), was used to perform the fusion of EEG and fNIRS features (Al-Shargie et al., 2016). Some researchers also used deep learning-based feature fusion approaches, such as tensor fusion and *pth*-order polynomial fusion (Chiarelli et al., 2018; Sun et al., 2020). These multi-modal fusion approaches have been able to improve the accuracy, but at the cost of increasing computational complexity and decreasing stability. In Yin et al. (2015), the authors introduced a features combination and optimization approach using joint mutual information (JMI), and the study decoded the motor imagery of the force and speed of hand clenching. The feature optimization method, JMI, was developed with the intention to remove unessential information that may reduce classification accuracy. The authors reported achieving an improved performance of up to 5% when compared to previous studies. In 2017, Al-Shargie et al. (2017) proposed a canonical correlation analysis (CCA) to perform feature-based fusion. The aim was to investigate the effects of mental stress on prefrontal cortex (PFC) based upon simultaneously recorded EEG and fNIRS signals. CCA is a statistical method that maximizes the correlation between the features of brain signals recorded by each modality EEG-fNIRS.

Though the improvements achieved by jICA, JMI, and CCA were satisfactory, the fusion was applied on the feature level, where the two modalities were processed separately. Therefore, a true system-level fusion is needed in order to capture the maximum benefits of the hybrid BCI, maximize the correlation between each modality, and reduce the computational complexity. In this study, we propose a novel hybrid BCI fusion approach using Multi-resolution singular value decomposition (MSVD) to perform a feature-based and system-based fusion for both EEG and fNIRS by employing selected channels from each hemisphere. The MSVD has previously been utilized primarily for image analysis, fusion (Kakarala and Ogunbona, 2001; Ashin et al., 2005; Naidu, 2011) and pattern recognition (Lung, 2002). To our knowledge, the present study is the first attempt to perform a hybrid EEG-fNIRS BCI fusion at the system level using MSVD. This approach not only helps to improve the classification accuracy, but also to reduce the dimensionality and the computational complexity. To evaluate the performance, the proposed approach is tested for two datasets: Buccino dataset (Buccino et al., 2016) and dataset from Technical University Berlin (TU Berlin) (Shin et al., 2018b).

## 2. Materials and Methods

### 2.1. Datasource and Experimental Paradigm

The proposed approach has the tendency to work with datasets of different nature. To prove its effectiveness, it is tested on two simultaneously recorded EEG-fNIRS data for motor execution and cognitive tasks. Both datasets have been widely used by the research community in the recent past as they can be openly accessed (Congedo et al., 2017; Saadati et al., 2020).

#### 2.1.1. Buccino dataset

The publicly available dataset obtained from an online repository (http://dx.doi.org/10.6084/m9.figshare.1619640 and http://dx.doi.org/10.6084/m9.figshare.1619641) was provided by Buccino et al. (2016). The raw data from EEG and fNIRS was concurrently recorded for four motor execution tasks– right and left arm; right and left hand–against the rest. Fifteen healthy subjects, aged between 23 and 54, were involved in the experiments that lasted an hour. A screen was installed nearly 100 cm away from the subjects on which visual instructions were displayed; the subjects were asked to follow the instructions without any intentional delay. The total duration of each experiment was segmented into rest and activity periods; each trial started with a rest for 6 s followed by another 6 s of movements.

#### 2.1.2. TU Berlin Dataset

The second open-access dataset considered in this study was from TU Berlin (Shin et al., 2018b), where 26 healthy persons participated in the experiment ranging between 17 and 33 years of age. A 24in LCD monitor was placed in front of the participants, approximately at a distance of 1.2 m. They were instructed to place their middle and index fingers on the numeric keypad attached to the armrest of the chair. The EEG and NIRS signals were recorded simultaneously for three cognitive tasks over a period of approx. 3.5 h: n-back (0-, 2-, and 3-back), discrimination/selection response task (DSR), and word generation (WG). In this study, we considered only the n-back tasks where a series of nine tasks were performed by each participant. At the start of each series, a type of task is displayed on the screen for 2 s, followed by the actual task period of 40 s, and then 20 s rest period. The participants responded to the screen instructions by either pressing the target key (number 7) or non-target key (number 8) with their right index finger and right middle finger. More details about the dataset can be obtained from Shin et al. (2018b) and (http://doc.ml.tu-berlin.de/simultaneous_EEG_NIRS/).

### 2.2. Data Acquisition

#### 2.2.1. Buccino Dataset

The EEG system (microEEG, BioSignal Group, US) was used to record the signals through twenty-one channels, sampled at a rate of 250 Hz. The fNIRS system, NIRScout 8-16 (NIRx Medizintechnik GmbH, Berlin, Germany) equipped with 12 sources and 12 electrodes on 34 channels was used to acquire signals at a sampling frequency of 10.42 Hz. The EEG electrodes and fNIRS probes were mounted on an extended EEG cap (actiCAP 128, Brain Products GmbH, Germany) according to the international 10-20 system.

#### 2.2.2. TU Berlin Dataset

A multi-channel BrainAmp EEG amplifier (Brain Products GmbH, Gilching, Germany) working at a sampling rate of 1,000 Hz was used to store the raw EEG data. The fNIRS system, NIRScout (NIRx Medizintechnik GmbH, Berlin, Germany), combined with thirty-six channels was used to record data at a sampling rate of 10.4 Hz. Thirty EEG electrodes, and sixteen pairs of NIRS sources and detectors, were mounted on a cap (EASYCAP GmbH, Herrsching am Ammersee, Germany) according to the international 10-5 system.

### 2.3. Data Pre-processing

#### 2.3.1. Buccino Dataset

The initial trial was segmented out prior to the motor execution tasks. The raw fNIRS data obtained at a sampling frequency of 10.42 Hz was decomposed into Oxy-haemoglobin and Deoxy-haemoglobin concentration changes (HbO and HbR) through the Modified Beer-Lambert law (Cope et al., 1988; Baker et al., 2014). Later, the concentration signals were filtered with a 4th order IIR Butterworth filter between 0.01 and 0.2 Hz. The EEG signals were also filtered with a 4th order IIR Butterworth filter between 1 and 50 Hz to remove artifacts.

#### 2.3.2. TU Berlin Dataset

The raw NIRS data were transformed to HbO and HbR using the Modified Beer-Lambert law, and down-sampled to 10 Hz. The obtained data was filtered (6th order zero-phase Butterworth) with 0.2 Hz cut-off frequency to remove systemic physiological noises. The raw EEG data were down-sampled to 200 Hz and band-pass filtered (6th order zero-phase Butterworth) between 1 and 40 Hz. Additionally, the second-order blind identification method was applied to the filtered data to eliminate ocular artifacts.

The filtered EEG and fNIRS data were baseline-corrected by subtracting the mean and dividing by the standard deviation. For both datasets, the EEG data were downsized through an average moving window of 1 s to ascertain consistency and synchronization. Additionally, we selected HbO as the main feature for the fNIRS signal as the concentration change is more observable in HbO and can produce higher accuracy when compared to HbR and total haemoglobin (HbT) (Aihara et al., 2012; Morioka et al., 2014; Buccino et al., 2016).

### 2.4. Channel Selection

The criteria of channel selection is based upon the correlation coefficient, ρ, determined between the filtered data of each modality. Some researchers have investigated the utilization of the Pearson correlation coefficient to solve practical problems in medical industry (Yildiz and BERGIL, 2015; Akoglu, 2018). Our previous study in this context (Hasan et al., 2020) demonstrated that this approach can be effectively utilized to select optimal channels for EEG and fNIRS.

### 2.5. Feature Extraction

#### 2.5.1. Discrete Wavelet Transform (DWT)

**Figure 1:**
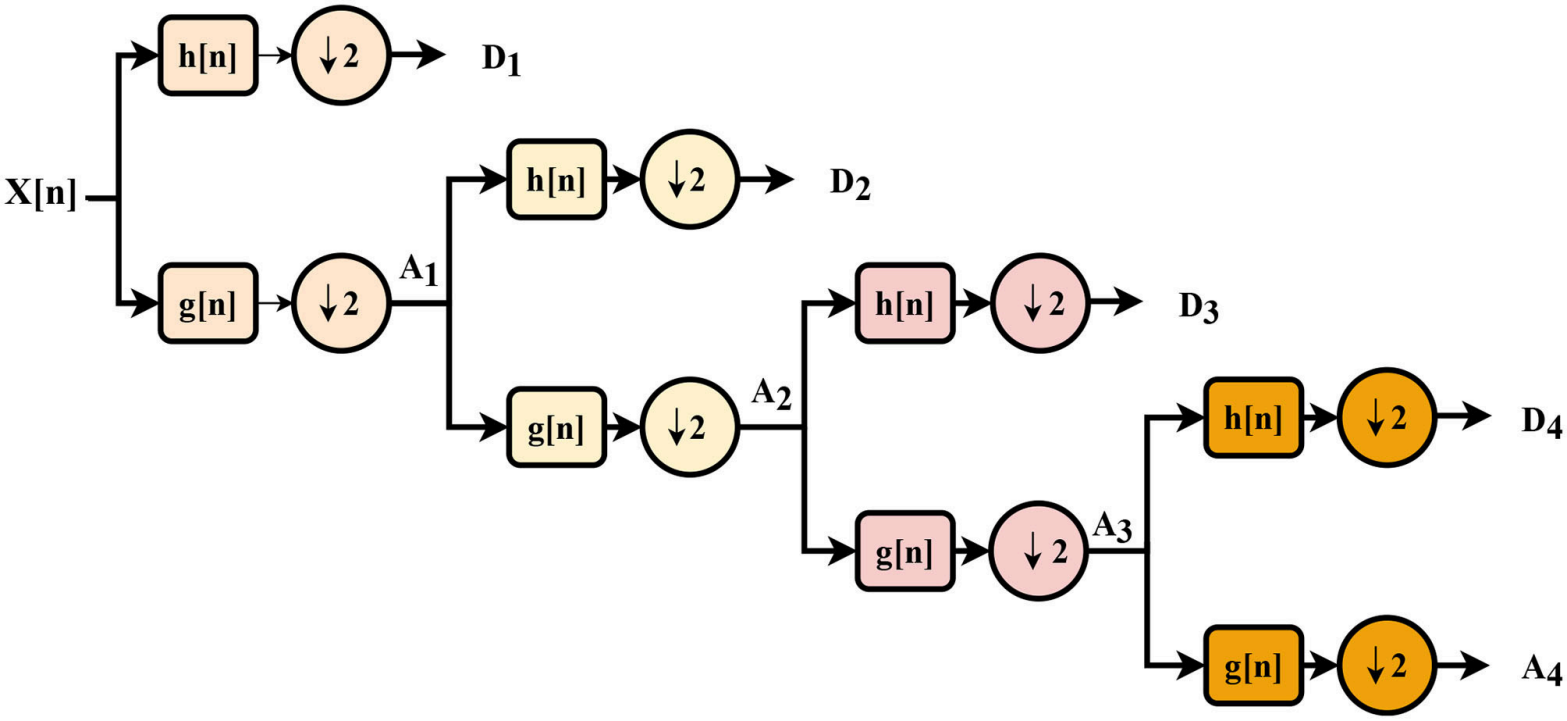
A four-level filter bank; h[n] is the high pass filter, g[n] is the low pass filter.

The DWT of a signal X[n], as shown in Figure 1, is obtained through a series of low- and high-pass filter pairs, named as quadrature mirror filters. As the frequency bandwidth is reduced to half, the filtered signal can be down-sampled by two according to the Nyquist's rule. The reduced output from the low- and high-pass filter branches are regarded as approximation (A_*i*_) and detail (D_*i*_) coefficients, where *i* represents the level of the transform. The same procedure can be repeated multiple times to improve the frequency resolution by considering the coefficients from the previous level as an input. The tree structure is also known as a filter bank. After each decomposition, the time resolution is halved through down-sampling, whereas the frequency resolution is doubled through filtering. Based upon the work of Subasi (2007), the authors in Li et al. (2017) reported that the approximation coefficient from the output of the last DWT layer is the main carrier of the signal's power. They suggested the use of a 4-layer “Symlet” wavelet network to obtain higher classification accuracy.

**Decomposition Level**

For DWT, the mother wavelet transform directly impacts the calculation of the approximation and detail coefficients (Mallat, 1989), thereby affecting overall accuracy. The most commonly used families are biorthogonal, reverse biorthogonal, daubechies, symlets, coiflets, discrete meyer, and haar (Faust et al., 2015). In this study, from the family of symelts, sym4 is selected having a filter size of eight as a mother wavelet.

The number of decomposition levels of DWT is associated with the input signal and mother wavelet. With more depth of decomposition, a detailed description of the signal can be obtained, but it may produce features redundancy leading to the lower accuracy and higher computational cost. The highest level *L* of the decomposition is determined as $floor\left(log_{2}\left(\frac{N}{F-1}\right)\right)$, where *N* is the size of the input signal and *F* is the mother wavelet filter size (eight in our case) (Wu et al., 2000). Chen et al. (2017) reported that beyond a certain level, not much improvement can be observed in the accuracy. Even in some cases, the accuracy even dropped with the increase in the decomposition level. Hence, more levels of decomposition do not necessarily mean improved accuracy, but definitely adding to the computational cost. For our case, we obtained the maximum accuracy with four levels of decomposition.

#### 2.5.2. Statistical Features

In addition to DWT, six different statistical features are extracted using spatial averaging of selected channels. The considered features set are: mean (M), peak (P), skewness (SK), kurtosis (KR), standard deviation (SD), and variance (VAR). The selection of these features is based upon the existing literature, where there is also a comparison between the performance of individual features and their combinations (Hong et al., 2017; Khan R. A. et al., 2018; Hasan et al., 2020). The extracted features set are re-scaled between 0 and 1, using:

(1) $$\begin{array}{l} X_{new}=\frac{X_{i}-\min\left(X_{i}\right)}{\max\left(X_{i}\right)-\min\left(X_{i}\right)} \end{array}$$

After processing the extracted features using Equation (1), the normalized feature vectors are obtained as M_*new*_, P_*new*_, SK_*new*_, KR_*new*_, SD_*new*_, and VAR_*new*_. To avoid ambiguity and for the sake of easiness, the normalized features are still represented using the same variables, but without the subscript.

## 3. Data-Fusion

### 3.1. Multi-Resolution Singular Value Decomposition (MSVD)

The motivation behind the proposed approach is to build a framework for multi-modal fusion using MSVD. Similar to DWT, an input signal is processed through high- and low-pass finite impulse response (FIR) filters at the first stage, followed by down-sampling. In the following stage, the approximation coefficient from the previous level is bifurcated to achieve decomposition (Naidu, 2011). The same procedure is repeated to obtain *d* level decomposition, where the FIR filters are replaced with the MSVD.

Let *X* ∈ ℝ^*n*′ × *m*′^ contains the statistical features of the input signal or the fused signal, where (*n*′, *m*′) are constrained as an even number due to the decomposition process.


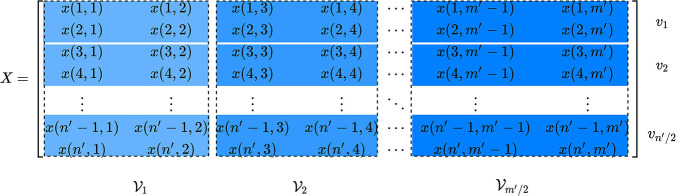


By introducing new variables (*n, m*) as $\left(\frac{n^{\prime }}{2},\frac{m^{\prime }}{2}\right)$, the data matrix, *A* ∈ R^4 × *nm*^, is built upon the matrix *X* as:

(2) $$\begin{array}{l} A=\left[\begin{array}{llll} V_{1} & V_{2} & \cdots & V_{m} \end{array}\right] \end{array}$$

where each $V_{i}$ contains two adjacent columns of *X*, and is defined as follows:

(3) $$\begin{array}{l} V_{i}=\left[\begin{array}{llll} \upsilon_{1} & \upsilon_{2} & \ldots & \upsilon_{n} \end{array}\right] \end{array}$$

Each individual vector υ_*i*_ contains a feature set of four elements of *X*, and is formulated as:

(4) $$\begin{array}{l} \upsilon_{\text{i}}={\left[\begin{array}{llll} \upsilon_{i}^{UL} & \upsilon_{i}^{UR} & \upsilon_{i}^{LL} & \upsilon_{i}^{LR} \end{array}\right]}^{T} \end{array}$$

where *UL*, *UR*, *LL*, and *LR* represents upper-left, upper-right, lower-left, and lower-right elements, respectively.

Afterwards, the singular value decomposition is applied on the generated data matrix *A* as:

(5) $$\begin{array}{l} A=USV^{T} \end{array}$$

where the columns of *U* contain left singular vectors, *S* holds singular values as diagonal entries, and rows of *V*^*T*^ have the right singular vectors. The singular vectors are chosen to satisfy:

(6) $$\begin{array}{l} U^{T}A=SV^{T} \end{array}$$

A scatter matrix, *T* ∈ R^4 × *nm*^, is defined using Equation (6) as:

(7) $$\begin{array}{l} T=U^{T}A \end{array}$$

The vectors $\left\{{\vec{t}}_{1},{\vec{t}}_{2},{\vec{t}}_{3},{\vec{t}}_{4}\right\}$ specify the rows of *T*, where each ${\vec{t}}_{i}\in {\mathbb{R}}^{1\times nm}$. These vectors are reshaped to generate corresponding matrices {Γ_1_, Γ_2_, Γ_3_, Γ_4_}, where each $\Gamma_{i}\in {\mathbb{R}}^{n\times m}$. A split matrix φ ∈ ℝ^*n*′ × *m*′^ is introduced as:


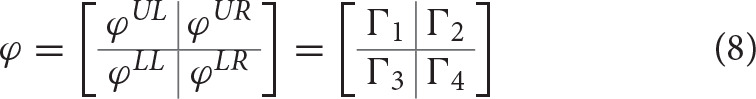


**Figure 2:**
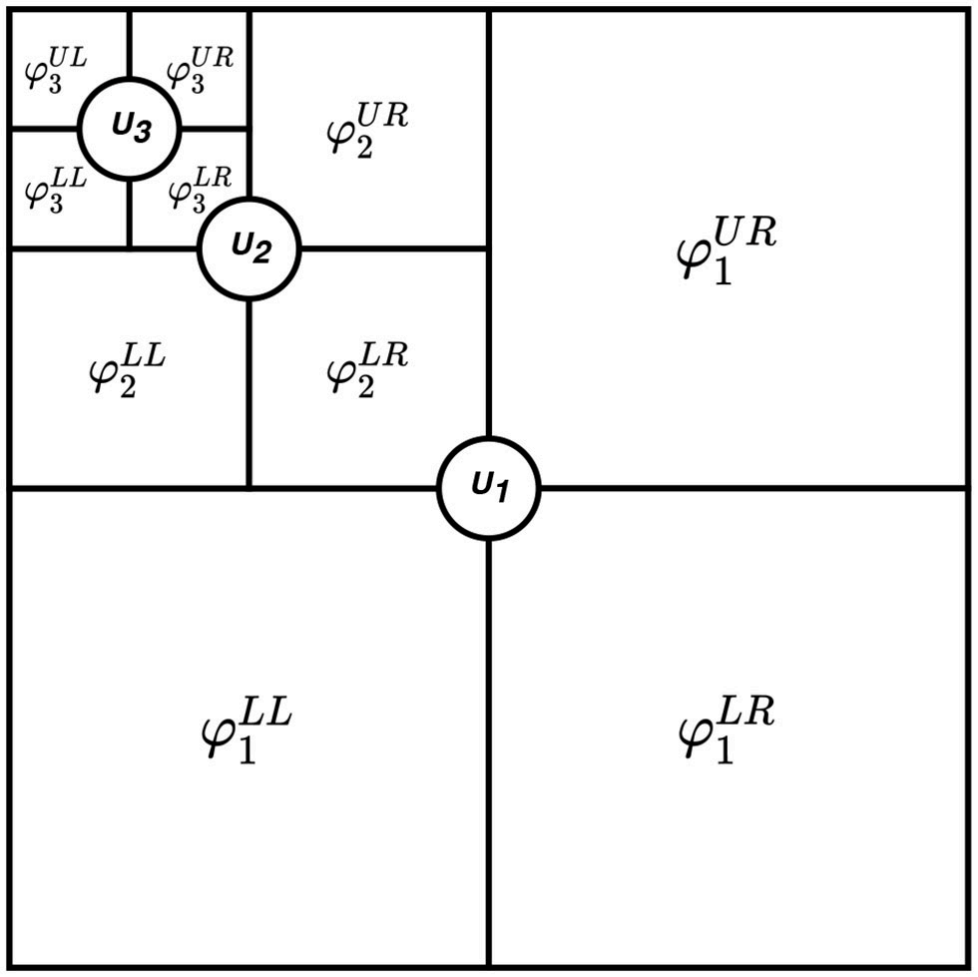
A three-level multi-resolution decomposition structure.

**Figure 3:**
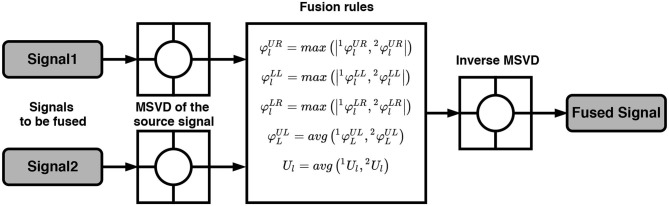
MSVD fusion scheme.

Figure 2 shows the structure of the split matrix with three decomposition levels. In case of a multiple input signals, a split matrix using MSVD is obtained for an individual input. For instance, two input signals *S*_1_ and *S*_2_, having the same dimensions, are decomposed into *L* (*l* =1,2,., *L*) levels using MSVD (Figure 3). After the generation of the split matrix, fusion has to be performed. To do so, it is necessary to store detail component vectors $\varphi_{l}^{\left\{UR,LL,LR\right\}}$ and singular-vector matrix *U*_*l*_ for *l* =1,2,., *L*, whereas it is sufficient to store the approximation component vector only at the coarest level *L* i.e., $\varphi_{L}^{\left\{UL\right\}}$. The fusion rules mentioned in Figure 3 are used to fuse the signals from multi-sources. At each decomposition level *l*, the largest absolute detail component vector is selected since it assumed to carry the main power of the signals. Similarly, the average of the singular-vector matrix is computed at each level. At the coarest level (*l* = *L*), the average of the approximation coefficients is calculated.

It can be observed that based upon the information from the split matrix, a decision is made. Once the fusion rules are applied in order to merge all the information into a single modality, an inverse process is applied to obtain the fused matrix.

The scatter matrix *T* is reconstructed based upon the information from the split matrix since the steps are reversible. The sub-matrices of the split matrix φ are reshaped from ℝ^*n*×*m*^ → ℝ^1 × *nm*^ to redefine scatter matrix *T*:

(9) $$\begin{array}{l} T=\left[\begin{array}{c} {\vec{t}}_{1}\\ {\vec{t}}_{2}\\ {\vec{t}}_{3}\\ {\vec{t}}_{4} \end{array}\right] \end{array}$$

Using Equation (9), a data matrix *A* is obtained as:

(10) $$\begin{array}{l} A=UT \end{array}$$

The structure of the data matrix is defined as:


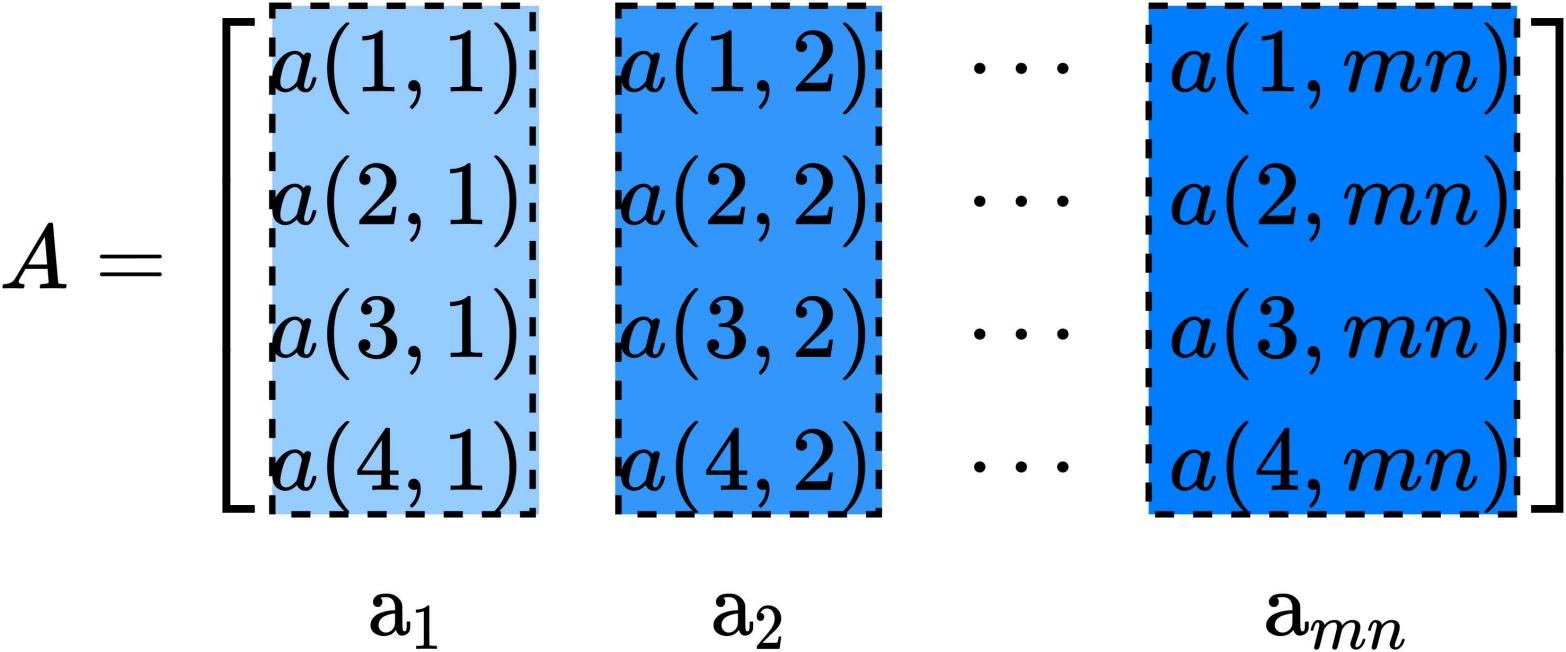


Each column vector a_*i*_ contains four elements and is used to define a fused feature matrix *X* as:

(11) $$\begin{array}{l} X=\left[\begin{array}{ccccc} {\text{a}}_{1} & {\text{a}}_{m+1} & \ldots & {\text{a}}_{m\left(n-2\right)+1} & {\text{a}}_{m\left(n-1\right)+1}\\ {\text{a}}_{2} & {\text{a}}_{m+2} & \ldots & {\text{a}}_{m\left(n-2\right)+2} & {\text{a}}_{m\left(n-1\right)+2}\\ \vdots & \vdots & \ddots & \vdots & \vdots \\ {\text{a}}_{m-1} & {\text{a}}_{2m-1} & \ldots & {\text{a}}_{m\left(n-1\right)-1} & {\text{a}}_{mn-1}\\ {\text{a}}_{m} & {\text{a}}_{2m} & \ldots & {\text{a}}_{m\left(n-1\right)} & {\text{a}}_{mn} \end{array}\right] \end{array}$$

where

(12) $$\begin{array}{l} {\text{a}}_{i}=\left[\begin{array}{cc} a\left(1,i\right) & a\left(2,i\right)\\ a\left(3,i\right) & a\left(4,i\right) \end{array}\right] \end{array}$$

### 3.2. Feature-Based Fusion

**Figure 4:**
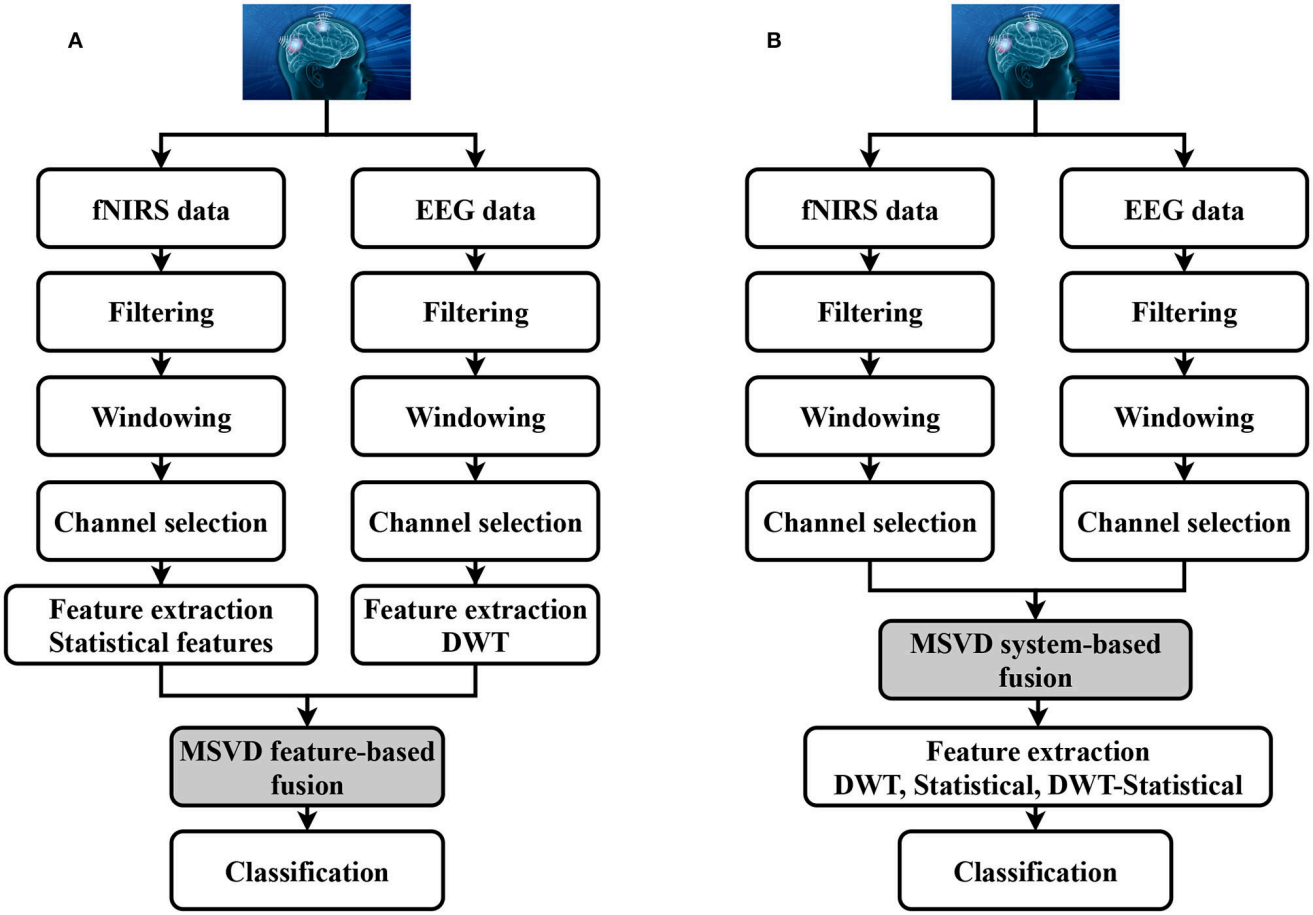
Hybrid BCI system using **(A)** Feature-based fusion. **(B)** System-based fusion.

EEG-fNIRS correlation analysis helped to reveal the intrinsic relationship between both modalities. To maximize the accuracy and to increase the number of the generated commands, statistical and optimization-based feature extraction methods are among the most commonly used. However, most of the previous studies focused on the feature-based level fusion by simply concatenating EEG and fNIRS features [*f*_*EEG*_ : *f*_*FNIRS*_]. In this paper, we proposed the utilization of the MSVD to perform EEG-fNIRS feature-based fusion. For the given datasets, the details about the pre-processing steps, such as filtering and windowing are provided in section 2.3. Based upon the correlation coefficient, six optimal channels are selected from both modalities. Six statistical features from the fNIRS and six normalized DWT features (one from each channel) from the EEG are extracted (Figure 4A). Afterwards, MSVD decomposes the features set into sub-bands through filtering, and the output of each filter is dismantled by a factor of two to complete the first level of decomposition. Afterwards, the fusion rules mentioned in Figure 3 are applied, followed by the classification to determine the specific tasks.

### 3.3. System-Based Fusion

Figure 4B is a schematic representation of the proposed system-based fusion using MSVD for a hybrid BCI system. To our knowledge, none of the previous studies have so far applied fusion at the system-level, mainly due to the complexity and lack of computational approaches. The pre-processed signals (filtered, down-sampled) are used to extract the desirable number of channels using the correlation coefficient (Hasan et al., 2020). Six channels are selected from both modalities and processed using MSVD to perform system-based fusion. Multiple features are extracted from the fused signal: DWT, statistical, and a combination of DWT and statistical. Later, the extracted features are fed to the classifier to determine the specific tasks.

## 4. Results

This section mainly evaluates the performance of the two fusion schemes, feature- and system-based fusion, by utilizing Buccino and TU Berlin datasets. To reduce the computational complexity, a reduced number of channels of both modalities are utilized for the classification. On Buccino dataset, the computational time, recorded as the temporal distance between the filtration and feature extraction, is highlighted in Table 1 for EEG and fNIRS based upon all channels and the reduced number of channels. The analysis made is based upon the sampled data of 1 s, obtained through both modalities. The response time is reduced by 40 and 50% for EEG and fNIRS, respectively.

**Table 1:** Impact of channels selection on the computational time for both EEG and fNIRS.

**Number of channels**	**EEG (s)**	**fNIRS (s)**
All channels	0.1639	0.1405
6 channels	0.0935	0.0724

### 4.1. Classification

For EEG and fNIRS, the classification accuracies using the KNN and Tree classifiers are evaluated for four different motor tasks against the rest based upon Buccino dataset; whereas, hybrid EEG-fNIRS analysis is made for both Buccino and TU Berlin datasets, using the same classifiers. The KNN classifier proximate the nearest observation points from the training data into a single class. It is preferred due to its simplicity, easiness to implement and high classification performance (Bablani et al., 2018). The Tree classifier constructs the decision tree with branches and node(s) based on the extracted features. At each node, either a single feature or several features contribute to minimizing the entropy label of the class (Aydemir and Kayikcioglu, 2014). To evaluate the classifiers' performance, a 10-fold cross-validation scheme is applied to the feature vectors of EEG, fNIRS, and EEG-fNIRS.

### 4.2. EEG

The average classification accuracies in Table 2 are obtained using the KNN and Tree classifiers for the eight selected subjects based upon Buccino dataset. The four approximation coefficients obtained using four-level DWT are defined as the EEG features. It is noted that the Tree classifier, when compared to KNN, has only been able to produce moderate results. The classification accuracy of more than 80% is achieved using the last approximation coefficient A_4_, when processed through KNN. This phenomenon is observed as DWT helps decompose the EEG signals into four layers, and the last approximation coefficient is assumed to hold the most effective event-related potential (ERP) of the brain activity. The DWT decomposition also helped to reduce the dimensionality of the system.

**Table 2:** Average classification accuracies for the eight subjects using the EEG.

**Features set**	**EEG**	
	**KNN (%)**	**Tree (%)**
A_1_	44.45	50.62
A_2_	59.96	53.10
A_3_	70.83	60.55
A_4_	82.36	71.32

### 4.3. fNIRS

Table 3 shows the KNN and Tree classification results obtained for the eight subjects (Buccino dataset) using fNIRS-only features. A feature set, consisting of fifteen feature vectors, is developed from a combination of the six statistical features. In this study, it is revealed that the combination of mean and skewness produces the highest average classification accuracies for both KNN and Tree. It is concluded that, similar to EEG, fNIRS has not been able to produce any satisfactory results.

**Table 3:** Average classification accuracies for the eight subjects using the fNIRS.

**Features set**	**fNIRS**	
	**KNN (%)**	**Tree (%)**
M, P	64.80	69.82
M, SK	70.97	73.63
M, KR	68.85	71.81
M, SD	69.62	71.66
M, VAR	68.08	71.66
P, SK	70.36	73.27
P, KR	66.42	71.32
P, SD	67.58	71.13
P, VAR	67.28	71.10
SK, KR	56.66	64.52
SK, SD	65.00	69.16
SK, VAR	64.11	69.13
KR, SD	63.81	68.36
KR, VAR	62.98	68.38
SD, VAR	46.91	56.61

### 4.4. Hybrid EEG-fNIRS

The performance of the hybrid EEG-fNIRS based upon feature- and system-based fusion is evaluated using accuracy, specificity, and area under curve (AUC). The most commonly used performance measures, such as precision, recall, and F1-score are not useful for the multi-classification, as they produces the same results. Hence, for the purpose, AUC is employed, its value ranges between 0 and 100%. The closer the value is to 100%, the better is the classification performance of the model.

#### 4.4.1. Feature-Based Fusion

For feature-based fusion, based upon the selected channels from both modalities, the six statistical features from the fNIRS, and DWT's last layer approximation coefficients from the EEG are used as the main features. The number of selected channels from both modalities is kept the same. A combined feature set of EEG-fNIRS is processed through MSVD. Table 4 illustrates the classification performance measures obtained for the eight subjects using feature-based fusion.

**Table 4:** Classification performance of the hybrid EEG-fNIRS for the eight subjects using the (Tree,KNN), based upon feature-based fusion.

	**S1/S2/S3/S4**			**S5/S6/S7/S8**		
	**Accuracy (%)**	**Specificity (%)**	**AUC (%)**	**Accuracy (%)**	**Specificity (%)**	**AUC (%)**
Buccino	(72.1,93.6)	(80.0,93.6)	(81.0,94.0)	(78.9,97.0)	(93.4,97.0)	(86.0,97.0)
TU Berlin	(87.4,94.4)	(88.8,94.7)	(90.0,95.0)	(83.4,95.5)	(91.7,97.0)	(93.0,98.0)
Buccino	(73.2,85.7)	(81.0,85.0)	(82.0,89.0)	(70.9,85.0)	(78.0,87.3)	(85.0,89.0)
TU Berlin	(71.0,72.4)	(77.5,74.0)	(81.0,76.0)	(68.6,76.4)	(76.1,79.0)	(80.0,83.0)
Buccino	(79.1,92.4)	(81.3,92.0)	(83.0,92.0)	(77.7,95.8)	(81.0,98.0)	(88.0,98.0)
TU Berlin	(84.8,92.1)	(86.8,93.2)	(91.0,94.0)	(72.4,79.9)	(78.4,83.5)	(87.0,86.0)
Buccino	(77.1,86.1)	(85.3,89.0)	(87.0,91.0)	(70.9,86.4)	(72.1,85.0)	(74.0,85.0)
TU Berlin	(77.0,91.0)	(81.3,91.9)	(87.0,93.0)	(78.7,81.9)	(82.5,84.7)	(91.0,90.0)

**Buccino Dataset**

The proposed method delivered promising performance for the motor execution tasks. Table 4 shows consistent accuracy above 85% across all subjects using the KNN. Considering all the subjects, the average classification accuracies of 90.25 and 74.98% are obtained through the KNN and Tree classifiers, respectively. It can be observed that the KNN has been able to outperform the Tree classifier for feature-based fusion. It is also noticeable that there is a variation among individual subject's performance, causing a direct effect on the overall accuracy. There can be many possible reasons for this phenomenon: it could be due to the subject's experiencing such tasks first hand or loss of interest at some stage during the process. This can be corrected by properly training the subjects before performing the experiments, as well as by shortening the duration of the experiments. Regarding the individual's performance, the best performing subjects are S5 and S7, who achieved the highest accuracies of 97.0 and 95.8% through KNN.

**TU Berlin Dataset**

The n-back tasks classification results using the KNN and Tree classifiers are presented in Table 4. For all the subjects, highest classification accuracy is achieved by the KNN. The highest accuracies (%) attained for the eight subjects are 94.4, 72.4, 92.1, 91.0, 95.5, 76.4, 79.9, and 81.9. The average classification accuracies obtained using the KNN and Tree classifiers are 85.45 and 77.91%, respectively. It is re-observed that due to the individual's performance, there has been a significant drop in the overall accuracy, despite the fact that four subjects have been able to achieve an accuracy of 91% or above (KNN). Although, the results are reported for a single feature set (DWT-statistical), the proposed method can be further tested with other combinations to yield the highest accuracies.

#### 4.4.2. System-Based Fusion

System-based fusion presents many advantages as compared to feature-based fusion; it is less time-consuming since we are analyzing the fused signals instead of processing each signal separately, and then fusing them. It is also more robust toward cross-data set variations of the components, which can be used for generating group-level inferences in different ways. The processed EEG-fNIRS data obtained from the selected channels is fused using MSVD system-based Fusion.

**Buccino dataset**

Table 5 summarizes the classification accuracies obtained using a combined features set (DWT, statistical, DWT-statistical) through the KNN and Tree classifiers for the eight subjects. Among three features set, DWT is able to produce the highest accuracy of 98.9% (KNN) followed by DWT-statistical which attained 94.43% (Tree) at most. Moreover, consistent best accuracies (%) were 97.0, 98.9, 98.9, 98.6, 93.6, 98.3, 98.2, 98.9 for eight subjects, respectively, as obtained using the KNN. Based upon the performance, S2, S3 and, S8 can be considered as the best-performing subjects.

**Table 5:** Classification performance of the hybrid EEG-fNIRS for the eight subjects using the (Tree,KNN), based upon system-based fusion.

		**S1/S2/S3/S4**			**S5/S6/S7/S8**		
	**Features set**	**Accuracy (%)**	**Specificity (%)**	**AUC (%)**	**Accuracy (%)**	**Specificity (%)**	**AUC (%)**
Buccino	Six Statistical and DWT	(81.1,65.1)	(94.1,89.6)	(93.0,80.0)	(82.1,63.5)	(92.0,69.0)	(94.0,72.0)
	Six Statistical	(50.1,41.6)	(85.7,83.7)	(57.0,52.0)	(53.0,44.3)	(58.3,52.1)	(61.0,59.0)
	DWT	(83.1,97.0)	(94.7,99.0)	(93.0,97.0)	(83.8,93.6)	(88.2,98.1)	(94.0,99.0)
TU Berlin	Six Statistical and DWT	(95.6,83.4)	(95.7,84.6)	(96.0,81.0)	(96.1,79.5)	(98.5,82.0)	(99.0,89.0)
	Six Statistical	(62.4,59.7)	(66.0,71.3)	(68.0,60.0)	(46.1,45.5)	(62.0,60.0)	(66.0,63.0)
	DWT	(96.7,99.5)	(96.7,99.9)	(97.0,100)	(96.5,96.6)	(98.7,99.0)	(99.0,100)
Buccino	Six Statistical and DWT	(93.8,90.6)	(98.0,96.9)	(95.0,92.0)	(91.7,79.6)	(96.3,81.2)	(98.0,82.0)
	Six Statistical	(59.8,53.0)	(61.0,54.2)	(71.0,65.0)	(54.5,51.7)	(62.0,59.2)	(62.0,61.0)
	DWT	(94.3,98.9)	(95.1,99.0)	(95.0,99.0)	(92.4,98.3)	(98.0,99.0)	(98.0,99.0)
TU Berlin	Six Statistical and DWT	(94.6,80.7)	(94.9,83.9)	(95.0,84.0)	(95.6,82.6)	(97.0,88.0)	(98.0,89.0)
	Six Statistical	(51.3,50.2)	(61.2,58.0)	(63.0,59.0)	(54.6,56.0)	(65.0,56.0)	(67.0,73.0)
	DWT	(95.9,99.7)	(96.9,99.8)	(97.0,100)	(95.9,99.4)	(97.7,99.8)	(98.0,100)
Buccino	Six Statistical and DWT	(94.4,87.4)	(95.9,90.9)	(94.0,84.0)	(91.8,77.1)	(93.9,79.0)	(94.0,86.0)
	Six Statistical	(73.8,71.2)	(81.5,79.8)	(58.0,54.0)	(54.9,47.4)	(60.0,55.0)	(61.0,56.0)
	DWT	(95.0,98.9)	(96.3,99.2)	(95.0,99.0)	(92.4,98.2)	(95.0,98.0)	(95.0,98.0)
TU Berlin	Six Statistical and DWT	(96.2,81.8)	(97.8,84.0)	(98.0,86.0)	(93.4,84.7)	(98.0,88.0)	(98.0,90.0)
	Six Statistical	(56.4,54.5)	(59.0,62.0)	(70.0,65.0)	(59.1,56.8)	(75.0,60.0)	(79.0,71.0)
	DWT	(96.0,99.5)	(97.0,99.0)	(98.0,100)	(96.9,99.3)	(98.8,99.9)	(99.0,100)
Buccino	Six Statistical and DWT	(89.3,85.0)	(94.9,90.0)	(94.0,91.0)	(91.2,78.1)	(94.7,79.0)	(95.0,86.0)
	Six Statistical	(56.3,52.6)	(62.0,56.0)	(63.0,57.0)	(55.2,50.7)	(62.0,55.9)	(63.0,56.0)
	DWT	(92.0,98.6)	(96.2,99.0)	(96.0,99.0)	(91.9,98.9)	(96.0,99.0)	(96.0,100)
TU Berlin	Six Statistical and DWT	(77.7,79.5)	(85.0,83.0)	(87.0,84.0)	(94.0,76.8)	(96.5,76.0)	(97.0,83.0)
	Six Statistical	(63.3,63.5)	(66.4,64.0)	(76.0,71.0)	(40.9,40.9)	(59.0,52.0)	(61.0,57.0)
	DWT	(79.6,80.1)	(85.0,81.0)	(88.0,82.0)	(95.5,99.3)	(97.0,99.6)	(98.0,100)

**TU Berlin Dataset**

In Table 5, performance measures based upon the classification results are shown. The highest and the lowest accuracies of 99.7 and 40.9% are obtained using DWT and the six statistical features, respectively. The huge difference between the best-performing and worst-performing subjects causes the significant drop in the overall accuracy. Therefore, extreme caution must be taken to exclude the non-favorable features and subjects. For eight subjects, KNN in comparison to Tree has been able to produce the highest accuracies (%) of 99.5, 99.7, 99.5, 80.1, 96.6, 99.4, 99.3, and 99.3.

## 5. Discussion and Conclusion

A hybrid EEG-fNIRS BCI enables the assessment of brain activities from different perspectives; hence, a broader range of information is obtained. Additionally, it also compensates for the weaknesses of individual modalities. The performance of the hybrid EEG-fNIRS is compared against EEG-only and fNIRS-only for the eight subjects. The results supported the argument that the hybrid EEG-fNIRS should be preferred over the individual modalities. The obtained classification accuracy for the hybrid system is higher than EEG-only and fNIRS-only for all subjects. The reduced number of channels from both modalities are utilized to obtain the results. The selected channels are based upon the ranking of the correlation coefficient; the six highest ranked channels of both modalities are selected. As shown in Table 1, the response time is improved by 40% for both modalities without affecting the accuracy.

In this study, we presented an MSVD approach for bi-modalities data-fusion. The proposed approach is investigated for both feature- and system-based fusion of EEG-fNIRS, with the intention to improve the system's performance and to reduce dimensionality. The MSVD-based data-fusion works on the same principle as DWT; at each level, the signals are filtered and dismantled by a factor of two to decompose the data into their latent components. From the classification performance results in Tables 4, 5, it is apparent that the system-based fusion dominated the feature-based fusion for the all the subjects from both datasets using the Tree classifier. Contrarily, KNN has performed better for the feature-based fusion rather than the system-based fusion in most cases. Overall, the results show that MSVD is a powerful tool that naturally allows for the analysis and fusion of multiple data sets. Being quite simple from the computational perspective, it could be well-suited for real-time applications as well.

The analysis and results are obtained from offline data, but the proposed approach is implementable for a real-time setup. Instead of processing all the channels from both modalities, only the most optimized channels using a correlation coefficient can be applied for feature extraction. It is shown in Hasan et al. (2020) that it helps to reduce the computational burden while maintaining the classification accuracy. The selection of channels for the individual subjects can be added as an initialization step. The computation of SVD for a large matrix can be time-consuming; hence, limits the real-time application. For a rectangular matrix, instead of computing the SVD of a matrix *A* as in Equation (5), we can form a square matrix i.e., *A*^*T*^*A* for a thin matrix, and *AA*^*T*^ for a fat matrix to compute the SVD. The computation of SVD for these square matrices is considered efficient; therefore, suitable for online systems.

The features selection does have direct impact on the classification accuracy; thus, care must be taken in this regard. It is desirable that those features must be extracted, who truly represent the data and are as compact as possible. In Table 5, three different feature sets–DWT features, six statistical features, and a combination of DWT-statistical–are extracted to evaluate the performance of the system-based fusion for a hybrid EEG-fNIRS. In contrast to the feature-based fusion approach, the features are extracted from the fused EEG-fNIRS signal in the system-based fusion. Thus, for both fusion schemes, different behaviors can be expected. On Buccino dataset, the results show that the features set pertaining to the DWT-statistical, statistical, and DWT, in case of the KNN (Tree) classifiers, have 78.3% (89.4%), 51.6% (57.2%), and 97.8% (90.61%) average accuracies for all the subjects, respectively. On TU Berlin dataset, the average accuracies obtained for all the subjects, using the KNN (Tree) classifiers, for features set related to DWT-statistical, statistical, and DWT are 81.12% (92.9%), 53.38% (54.26%), and 96.67% (94.12%), respectively. These numbers reveal that by using the last layer's approximation coefficient of DWT, the highest accuracy is achieved; whereas, the lowest accuracy is obtained using the six statistical features. For DWT-statistical and statistical features, Tree classifier yielded the highest average accuracies; whereas, KNN achieved the highest accuracy for DWT features.

System-based fusion using MSVD enables the processing of fused EEG-fNIRS signals, rather than processing each modality separately for feature extraction and fusing them later. One of the concerns of this study, when it comes to system-based fusion, is the requirement of the same number of channels for both modalities, thus making channel selection compulsory. As such, future work will explore the possibility of system-based fusion when there is a mismatch between the number of channels for both modalities.

The second limitation of our study is the manual selection of features for the classification. The manual extraction of the features is a cumbersome process and has a direct impact on the classification accuracy. With the selection of optimal features, effective pre-processing, and various classification techniques, this accuracy can be improved (Khan R. A. et al., 2018; Hasan et al., 2020). However, it is not guaranteed that the optimal feature for one task will be able to produce desirable results for the other tasks. Therefore, this process has to be repeated for individual tasks, and this consumes a lot of time. Recently, deep learning techniques, such as convolution neural network (CNN) and recurrent neural network (RNN) have been utilized for automatic feature extraction, pre-processing, and classification (Zhang et al., 2017; Yang et al., 2018; Tayeb et al., 2019). The obtained results have been promising when compared to the conventional classifiers (Trakoolwilaiwan et al., 2017; Chiarelli et al., 2018; Kumar et al., 2019; Asgher et al., 2020; Ghonchi et al., 2020). Considering the improvement in accuracy obtained using deep learning techniques, even in light of the limited amount of data and fewer pre-processing requirements, this improvement motivates us to work upon the combination of such techniques with MSVD in the future.

In conclusion, the present work proposed a novel hybrid EEG-fNIRS fusion approach for the classification. The primary goal is to improve the classification accuracy and to reduce the computational complexity of the hybrid EEG-fNIRS BCI. In order to achieve this, an MSVD approach is proposed for feature-based fusion and system-based fusion. To validate the effectiveness of the proposed approach, eight different subjects were considered and multiple trials were performed. As is evident from the results, our hybrid system significantly reduces the computational burden while achieving higher classification accuracy. The authors anticipate and hope that the proposed fusion approach will lead to more effective applications of BCI.

## Data Availability Statement

Publicly available datasets were analyzed in this study. This data can be found here: http://dx.doi.org/10.6084/m9.figshare.1619641; http://dx.doi.org/10.6084/m9.figshare.1619640; http://doc.ml.tu-berlin.de/simultaneous_EEG_NIRS/.

## Ethics Statement

The studies involved were conducted according to the Helsinki declaration and were approved by the Institutional Review Board at University of Houston and by the Ethics Committee of the Institute of Psychology and Ergonomics, Berlin Institute of Technology. The patients/participants provided their written informed consent to participate in this study.

## Author Contributions

MK and MH conceived of the presented idea. MH developed the theory and performed the computations. MK verified the analytical methods. All authors discussed the results and contributed to the final manuscript.

## Conflict of Interest

The authors declare that the research was conducted in the absence of any commercial or financial relationships that could be construed as a potential conflict of interest.
